# Combined effects of fruit and vegetables intake and physical activity on the risk of metabolic syndrome among Chinese adults

**DOI:** 10.1371/journal.pone.0188533

**Published:** 2017-11-21

**Authors:** Xin-tong Li, Wei Liao, Hong-jie Yu, Ming-wei Liu, Shuai Yuan, Bo-wen Tang, Xu-hao Yang, Yong Song, Yao Huang, Shi-le Cheng, Zhi-yu Chen, Samuel D. Towne, Zong-fu Mao, Qi-qiang He

**Affiliations:** 1 School of Health Sciences, Wuhan University, P. R. China; 2 Ganzhou Health and Family Planning Committee, Ganzhou, P. R. China; 3 The first Clinical School, Wuhan University, Wuhan, P.R. China; 4 Department of Health Promotion and Community Health Sciences, Texas T&M Health Sciences Center, School of Public Health, College Station, TX, United States of America; INRA, FRANCE

## Abstract

**Background:**

Unbalanced dietary intake and insufficient physical activity (PA) have been recognized as risk factors for metabolic syndrome (MetS). We aimed to examine the independent and combined effects of fruit and vegetables (FV) intake and PA on MetS.

**Methods and findings:**

A cross-sectional survey was conducted among residents of China in 2009, with fasting blood samples collected. Participants were divided into sufficient/insufficient FV intake and adequate/ inadequate PA groups according to self-reported questionnaires. MetS was defined using the NCEP-ATPIII criteria. The difference of individual MetS components was compared across different PA or FV groups. Multivariable logistic regression was used to assess association between FV/PA and the risk of MetS. A total of 7424 adults were included in the current study. MetS was prevalent in 28.7% of participants, with 24.7% and 32.9% in male and female, respectively. Compared with those with inadequate PA and insufficient FV intake, participants with the combination of adequate PA and sufficient FV intake had the lowest risk of MetS (OR = 0.69,95%CI: 0.59–0.82), following by the group with adequate PA time but insufficient FV intake (OR = 0.74, 95%CI:0.65–0.83).

**Conclusion:**

Findings of the current study show that the combination of sufficient FV intake and adequate PA was significantly associated with reduced MetS risk among adult residents of China.

## Introduction

Metabolic syndrome (MetS), characterized by metabolic abnormalities including central obesity, dyslipidemia, hypertension, low high-density lipoprotein (HDL), and glucose intolerance, is associated with increased risk of cardiovascular diseases and type 2 diabetes[1]. It has been estimated that the prevalence of MetS in most countries around the world is between 20%-30% in adults[2]. MetS has been recognized as an important global public health issue. Thus it is essential to prevent MetS as well as to reduce the risk of related metabolic diseases.

Lifestyle factors including unbalanced dietary intake and inadequate physical activity (PA) have been recognized as risk factors of MetS. However, studies examining the association between dietary intake and MetS have showed inconsistent results [3–6]. Significant associations between high consumption of fruit and vegetables (FV) and lower risk of MetS as well as decreased blood pressure (BP) were founded in previous research[3]. There is also evidence showing that dietary intake with greater quantities of FV intake is associated with improvement in MetS and its components[4]. Nevertheless, a meta-analysis of eight randomized controlled trails found that FV interventions had no effects on improving the MetS components among patients[5]. Similarly, no associations were found between the amount of FV intake and the incident of MetS in a 9-year cohort study[6]. Beside FV intake, abundant PA has also been shown to reduce the risk of MetS. However, most studies focus on physical exercise rather than daily PA [7].

MetS is also a critical public health issue in China. The prevalence of MetS has nearly doubled from 13.7% in 2000 to 27.6% in 2009[8, 9]. Accompanied with the development of society and the economy, the lifestyles of residents of China also changed significantly within the same timeline. A significant decrease was found in the vegetables intake among Chinese adults, the average daily intake of vegetables dropped from 363g in 1993 to 321g in 2011, and the proportion of people who met the Chinese dietary guidelines of daily vegetable intake decreased from 58.8% in 1993 to 50.2% in 2011[10]. Further, research suggests a decline in PA among Chinese adults. It has been reported that PA decreased by approximately 42% from 1991 to 2011 among Chinese adults[11]. Therefore, it is necessary to explore the associations between these lifestyle factors and MetS. However, studies on the associations between FV consumption, PA and MetS are limited.

FV and PA are both simple and positive recommendation. Although some studies found weak effect of FV on the risk of MetS, others reported significant association between FV and MetS [3–6]. Furthermore, a healthy diet (especially FV consumption) and abundant PA have been widely recognized as two key strategies to reduce the risk of chronic diseases. Therefore, we examined the effects of PA and FV intake on the risk of MetS among a national sample of Chinese adults.

## Materials and methods

### Participants

The China Health and Nutrition Survey (CHNS), a nationwide longitudinal study, has been conducted since 1989. The CHNS measures health and related outcomes (e.g., nutrition, physical activity) among residents of China. Details of the survey have been described elsewhere[12]. In 2009 the CHNS began to include blood samples thereby allowing researchers to evaluate metabolic health outcomes. The 2009 CHNS sample was draw from a multi-stage random cluster sampling process, consisting of 216 communities from 9 provinces in China. All participants provided written informed consent. The study was approved by the institutional review board from the University of North Carolina-Chapel Hill and the China Center for Disease Control and Prevention.

A total of 7791 participants aged 18 years old and over with fasting blood samples were potentially eligible for inclusion in this study. Of these 7791 participants, 94 individuals were excluded because they did not have dietary information and an additional 261 participants were excluded for lacking anthropometric data including height, weight, and waist circumference. Further, 12 individuals who were pregnant at the time of measurement were excluded for analysis given pregnancy may affect dietary intake. The final analytical sample used in all analyses included 7424 adults.

### Measurements

Height, weight, and waist circumference (WC) were measured by trained interviewers. Body mass index (BMI) was calculated from weight and height. BP was measured three times by trained examiners using a mercury sphygmomanometer with mean values used in the current study. Venous blood samples were obtained by venepuncture after an overnight fast. All blood samples were collected in three vacuum tubes under standard protocol and processed within 3h. The fasting plasma glucose (FPG) and routine blood examination were measured in local hospitals. Other biochemical markers including HDL, triglyceride (TG) and total cholesterol (TC) were analyzed in the China-Japan Friendship Hospital (CJFH). Detailed laboratory analysis methods for the biomarkers have been described elsewhere[13].

Dietary intake was assessed by a combination of 24h food recalls on three consecutive days and a food inventory over the same 3-day period. For the 24h recall, food types, amounts, and consumption places were recorded by trained interviewers. FV included in this analysis was based on the classification of the “China Food Composition”[14], which classifies daily food and the nutrition components.

PA was estimated using self-reported questionnaires which recorded the time spent in leisure time activity, transportation, occupational and household chores, games or planned exercise. The questionnaire was similar to previous validated questionnaires [15, 16] and has been tailored to measure the PA that most Chinese adults engage in. Metabolic equivalents (METs) was used for measure the intensity of specific activity. A unit of MET is defined as the ratio work metabolic rate to a standard resting metabolic rate[17]. The METs of each activity in the questionnaire were reported in a previous research[18]. According to the CDC/ACSM, PA with corresponding METs between 3–6 is defined as moderate PA, and PA with corresponding METs over 6 is classified as vigorous PA [19]. Moderate-to-vigorous physical activity (MVPA) was calculated based on a combination of moderate minutes added to vigorous minutes, where vigorous minutes are multiplied by 2 [20].

### Covariates

Other sociodemographic variables were collected through questionnaires. Smoking status was classified as non-smoker, current smoker and former smoker. Regular alcohol consumption was classified as drinking alcohol at least once per week. Education level was categorized into three groups: illiterate and primary school, middle school, and high school or above.

### Metabolic syndrome

MetS was defined based on the updated the Adult Treatment Panel III of the National Cholesterol Education Program (NCEP-ATPIII)[21] as presenting with 3 or more of the following 5 components: 1) Abdominal obesity: WC ≥ 90 cm in men or ≥ 80 cm in women; 2) Hypertriglyceridemia: TG ≥1.7 mmol/L or antihypertensive medication; 3) Low HDL: HDL< 1.03 mmol/L in men or 1.3 mmol/L in women; 4) High BP: BP ≥ 130/85 mm Hg; 5) High fasting glucose: FPG ≥ 6.1 mmol/L or previously diagnosed type 2 diabetes.

### Statistical analysis

Participants were divided into sufficient /insufficient FV intake group according to the Chinese dietary guidelines, which recommend at least 500g FV intake per day for Chinese adults[10]. Given the absence of a recognized PA standard for Chinese adults, we used the dually recognized guidelines from the WHO [22] and US Centers for Disease Control and Prevention’s recommendation of engaging in at least 150 minutes MVPA/week to promote metabolic health and reduce the risk of NCDs among adults[22]. We, therefore, calculated the time spent on all MVPA per week. Participants were divided into adequate/inadequate PA groups according to the 150 mins MVPA/week WHO recommendation.

The individual MetS components and prevalence of MetS were compared across different PA or FV groups by covariance analysis or chi-square test, respectively. An interaction test was performed to assess the interactive effect of PA and FV on the risk of MetS. We divided subjects into four groups based on their PA and FV intake level, and explored the combined effects of PA and FV intake. Multivariable logistic regression was used to assess the association between PA, FV, and MetS, after adjusting for age, gender, total energy intake, area, married status, education level, smoking and alcohol status. Statistical analyses were performed using the SPSS statistical package (version 13.0; SPSS Inc, Chicago, Ill. USA).

## Results

Descriptive characteristics of the study population are summarized in Table 1. Overall, the average age of participants was 52 years with 66% of respondents residing in rural areas. Approximately 95% were married. Approximately 30% were current smokers (55.7% for males; 4.2% for females) and approximately 22% consumed alcoholic beverages at least once a week (40.5% for males; 3.8% for females). MetS was present in 28.9% of participants, with rates at 24.7% and 32.9% in males and females respectively. The most common issue facing male adults with MetS included high BP (42.4%), while the most common issue affecting female adults with MetS included central obesity (55.2%). Nearly a third (29.7%) of Chinese adults met the recommended Chinese dietary guidelines, while 50.2% of participants met the PA recommendation.

**Table 1:** Basic characteristics of the study population.

	Total (n = 7424)	Men (n = 3654)	Women (n = 3770)	P value
Age(years)	52.3±14.4	51.4±14.9	53.3±13.9	<0.001
BMI(kg/m^2^)	23.5±3.5	23.3±3.4	23.6±3.5	0.210
Rural area(%)	4932(66.4%)	2470(67.6%)	2462(65.3%)	0.040
Married	7061(94.5%)	3384(92.6%)	3632(96.3%)	<0.001
Smoking status				
Never	4973(67.0%)	14377(37.7%)	3696(95.4%)	<0.001
Current	2194(29.6%)	2035(55.7%)	159(4.2%)	
former	257(3.5%)	242(6.6%)	15(0.4%)	
Alcohol drinker (%)	1626(21.9%)	1481(40.5%)	145(3.8%)	<0.001
Education level				
low	3204(43.2%)	1195(32.7%)	2009(53.3%)	<0.001
middle	2466(33.2%)	1433(39.2%)	1033(27.4%)	
high	1754(23.6%)	1026(28.1%)	728(19.3%)	
SBP(mmHg)	125.9±19.1	126.3±17.6	125.4±20.5	<0.001
DBP(mmHg)	81.1±11.3	82.1±11.1	80.0±11.4	0.540
TG(mmol/L)	1.7±1.5	1.8±1.7	1.6±1.3	<0.001
HDL(mmol/L)	1.4±0.5	1.4±0.5	1.5±0.4	0.001
FPG (mmol/L)	5.4±1.6	5.4±1.6	5.3±1.3	<0.001
Sufficient FV intake (n,%)	2205 (29.7%)	1108(30.3%)	1097(29.1%)	0.250
Adequate PA (n, %)	3728(50.2%)	2147(58.8%)	1581(41.9%)	<0.001
MetS (n,%)	2142(28.9%)	903(24.7%)	1239(32.9%)	<0.001

Data are expressed as mean±SD. The adjusted mean levels of individual MetS components and prevalence of MetS according to PA levels and FV intake.

BMI: body mass index; WC: waist circumference; FPG: fasting plasma glucose; TG: triglycerides; HDL: high-density lipoprotein; SBP: systolic blood pressure; DBP: diastolic blood pressure; FV: fruits and vegetables; PA: physical activity; MetS: metabolic syndrome.

Sufficient FV intake: ≥ 500g FV intake daily. Adequate PA time: ≥ 150min moderate to vigorous PA per week.

Table 2 shows the prevalence of MetS and its components among PA and FV groups. Compared to those with inadequate PA levels, those with adequate PA showed significantly lower WC, SBP, TG, FPG and higher HDL, while lower prevalence of MetS, when compared with their counterparts. Although sufficient FV intake was associated with lower SBP and FPG, no other statistically significant difference was found between the two FV groups. Logistic regression models suggest that individuals with adequate PA had significantly lower risk for MetS (OR = 0.74, 95%CI:0.66–0.82) while those with sufficient FV intake had insignificantly lower risk for MetS (OR = 0.97,95%CI:0.86–1.09) in comparison with their counterparts.

**Table 2:** Difference of MetS components and the prevalence of MetS among different PA and FV groups.

	Inadequate PA (n = 3696)	Adequate PA (n = 3728)	Adjusted Difference(95%CI)*	P value	Insufficient FV (n = 5219)	Sufficient FV (n = 2205)	Adjusted Difference(95%CI)*	P value
MetS (%)	1279(34.6%)	863(23.1%)		<0.001	1525(29.2%)	617(28.0%)		0.287
MetS risk (OR, 95% CI)	Reference	0.74 (0.66,0.82)		<0.001	Reference	0.97 (0.86,1.09)		0.556
MetS components								
WC(cm)	82.40(0.38)	81.12(0.39)	1.08(0.59,1.56)	<0.001	81.60(0.37)	81.83(0.40)	-0.17(-0.68,0.34)	0.393
SBP(mmHg)	128.75(0.65)	127.47(0.68)	1.40(0.55,2.24)	0.003	128.43(0.65)	127.50(0.70)	0.95(0.07,1.84)	0.039
DBP(mmHg)	80.41(0.42)	80.16(0.43)	0.11(-0.43,0.65)	0.373	80.30(0.41)	80.30(0.45)	0.11(-0.56,0.58)	0.998
TG(mmol/L)	1.73(0.06)	1.56(0.06)	0.15(0.08,0.22)	<0.001	1.64(0.06)	1.62(0.06)	0.02(-0.06.0.10)	0.694
HDL(mmol/L)	1.47 (0.02)	1.50(0.02)	-0.03(-0.06,-0.01)	0.004	1.49(0.02)	1.48(0.02)	0.01(-0.01,0.04)	0.399
FPG (mmol/L)	5.39(0.06)	5.26(0.06)	0.13(0.06,0.20)	<0.001	5.35 (0.06)	5.26(0.06)	0.09(0.01,0.16)	0.028

WC: waist circumference; FPG: fasting plasma glucose; TG: triglycerides; HDL: high-density lipoprotein; SBP: systolic blood pressure; DBP: diastolic blood pressure; FV: fruits and vegetables; PA: physical activity; MetS: metabolic syndrome.

Sufficient FV intake: ≥ 500g FV intake daily. Adequate PA time: ≥ 150min moderate to vigorous PA per week.

*: Adjusted for age, gender, area, alcohol use, smoking status, education levels and energy.

In the interaction test, the term representing the interaction between PA and FV was not a significant predictor of MetS (P = 0.493). Nevertheless, as the main objective was to examine the individual and combined effects of PA and FV with MetS, the results were presented in Table 3. Compared with those with inadequate PA and insufficient FV intake, participants with adequate PA and sufficient FV intake had the lowest risk of MetS (OR = 0.69,95%CI: 0.59–0.82), followed by the group with adequate PA but insufficient FV intake (OR = 0.74, 95%CI:0.65–0.83).

**Table 3:** Combined associations of FV intake and PA with MetS.

	Inefficient FV intake		Sufficient FV intake	
PA	n(%)	OR(95% CI)	n(%)	OR(95% CI)
Inadequate	2715 (26.5%)	Ref	981 (31.6%)	1.02(0.90–1.19)
Adequate	2504 (20.9%)	0.74 (0.65–0.83)*	1224(22.5%)	0.69 (0.59–0.82)*

Results were adjusted for age, gender, area, alcohol use, smoking status, education level and energy.

* P <0.001

FV: fruits and vegetables; PA: physical activity; MetS: metabolic syndrome.

Sufficient FV intake: ≥ 500g FV intake daily. Adequate PA time: ≥ 150 min moderate to vigorous PA per week.

## Discussion

In the present population-based study, we examined the single and combined effects of engaging in adequate PA and sufficient FV intake on the risk of MetS. We found that adequate PA was significantly associated with lower MetS risk while sufficient FV intake was associated with some components of MetS. Furthermore, the combination of sufficient FV intake and adequate PA time were associated with the lowest risk of MetS.

Our findings indicate protective effects of PA on MetS risk and its components (i.e., WC, SBP, TG, etc.) were consistent with several previous studies[22]. For example, a cross-sectional study among Mexican-Americans found that adequate PA was associated with lower MetS risk[23]. However, these studies had relatively small sample sizes or only had a sample from single district[7, 22]. Other studies are mostly intervention studies in which vigorous, continuous exercises were assigned by researchers[24]. PA in our study consisted of occupational, transportation, household chores and leisure activity together, indicating that the benefits of PA may be accumulated from multiple settings in one’s daily life and not solely through planned exercise. Previous research indicated that the weekly volume (i.e. cumulative time per week), rather than frequency of PA sessions, was strongly associated with the MetS risk[25].

The effects of FV intake on MetS and its components were inconsistent in previous studies. In a 6 years prospective study, lower risk of MetS was observed among people following the Mediterranean diet, which including sufficient FV intake[26]. It has also been observed that adequate FV intake was associated with lower BP[27]. However, a cohort study showed no association between consumption of FV and the incidence of MetS[6]. In a meta-analysis of eight RCTs, Shin JY found that FV intake was only associated with a reduction in DBP, with no significant effect found for other MetS components[5]. These inconsistent findings might be explained by the diversity of FV intake across studies. It has been reported that green leafy vegetable intake rather than all types of vegetable intake were associated with decreased MetS risk[28]. Although significant associations were found only among sufficient FV consumption and SBP and glucose in our study, beneficial effects for FV combined with adequate PA were also observed. These results indicate that individuals with sufficient FV intake and adequate PA might have a lower likelihood of MetS, compared with those with inadequate PA and insufficient FV intake.

Identifying the exact mechanisms by which FV and PA reduce the risk of metabolic diseases needs further study. One possible explanation is that FV comprises relatively lower energy and higher fiber, which can help to reduce the overall energy content of daily dietary intake[29]. The decreased risk of MetS components with increasing consumption of FV intake may also be explained by the antioxidant content of FV which contribute to combat against free radicals and a reduction of systemic oxidative stress[30]. The lower glycemic index of FV may further explain the observed benefit of sufficient FV on glucose[3]. The influences of PA could be explained by its beneficial effects on body composition, including improving skeletal muscle insulin sensitivity and reduce insulin resistance[7]. Therefore, sufficient FV intake and PA can synthetically play a positive role in metabolic health.

### Limitations

The present study has several limitations. First, the cross-sectional nature of the survey prevents causal analyses, but rather the identification of associations between FV intake, PA and MetS. Second, the data about FV intake were collected by 3-day dietary recalls, which may be affected by recall bias and may also be inaccurate self-reported estimates given the difficulty to assess exact serving size of FV intake (e.g., participants were not trained in identifying serving sizes). Even so, the FFQ had been validated and widely used in previous studies[6, 31]. Third, the use of MVPA time per week to quantify adequate PA through self-reported questionnaires is not as accurate as using accelerometers. That said, the pragmatic nature of the survey and resources available prevented accelerometers from being widely used in the study area and therefore self-reported MVPA was used. Lastly, the implications of the current study must account for differences among ethnic, cultural, and lifestyle characteristics, given there may be difference among those in the current study and other populations.

### Conclusion

In conclusion, we observed that adequate PA was associated with lower MetS risk, whereas sufficient FV was slightly associated with some components of MetS but not significantly with MetS. The decreased prevalence of MetS in the group with sufficient FV and adequate PA was mainly explained by high PA. However, given that FVs are beneficial in preventing diseases, the combination of high FV and PA may concurrently improve metabolic health. Thus, multi-level strategies aimed at both factors are needed to reduce the burden of MetS.
